# Compromised white matter is related to lower cognitive performance in adults with phenylketonuria

**DOI:** 10.1093/braincomms/fcad155

**Published:** 2023-05-15

**Authors:** Raphaela Muri, Stephanie Maissen-Abgottspon, Murray Bruce Reed, Roland Kreis, Maike Hoefemann, Piotr Radojewski, Katarzyna Pospieszny, Michel Hochuli, Roland Wiest, Rupert Lanzenberger, Roman Trepp, Regula Everts

**Affiliations:** Department of Diabetes, Endocrinology, Nutritional Medicine and Metabolism, Inselspital, Bern University Hospital and University of Bern, 3010 Bern, Switzerland; Support Center for Advanced Neuroimaging (SCAN), University Institute of Diagnostic and Interventional Neuroradiology, Inselspital, Bern University Hospital and University of Bern, 3010 Bern, Switzerland; Graduate School for Health Sciences, University of Bern, 3012 Bern, Switzerland; Translational Imaging Center (TIC), Swiss Institute for Translational and Entrepreneurial Medicine, 3010 Bern, Switzerland; Department of Diabetes, Endocrinology, Nutritional Medicine and Metabolism, Inselspital, Bern University Hospital and University of Bern, 3010 Bern, Switzerland; Translational Imaging Center (TIC), Swiss Institute for Translational and Entrepreneurial Medicine, 3010 Bern, Switzerland; Department of Psychiatry and Psychotherapy, Medical University of Vienna, 1090 Vienna, Austria; Translational Imaging Center (TIC), Swiss Institute for Translational and Entrepreneurial Medicine, 3010 Bern, Switzerland; Magnetic Resonance Methodology, Institute of Diagnostic and Interventional Neuroradiology, Inselspital, Bern University Hospital and University of Bern, 3010 Bern, Switzerland; Translational Imaging Center (TIC), Swiss Institute for Translational and Entrepreneurial Medicine, 3010 Bern, Switzerland; Magnetic Resonance Methodology, Institute of Diagnostic and Interventional Neuroradiology, Inselspital, Bern University Hospital and University of Bern, 3010 Bern, Switzerland; Support Center for Advanced Neuroimaging (SCAN), University Institute of Diagnostic and Interventional Neuroradiology, Inselspital, Bern University Hospital and University of Bern, 3010 Bern, Switzerland; Translational Imaging Center (TIC), Swiss Institute for Translational and Entrepreneurial Medicine, 3010 Bern, Switzerland; Support Center for Advanced Neuroimaging (SCAN), University Institute of Diagnostic and Interventional Neuroradiology, Inselspital, Bern University Hospital and University of Bern, 3010 Bern, Switzerland; Department of Diabetes, Endocrinology, Nutritional Medicine and Metabolism, Inselspital, Bern University Hospital and University of Bern, 3010 Bern, Switzerland; Support Center for Advanced Neuroimaging (SCAN), University Institute of Diagnostic and Interventional Neuroradiology, Inselspital, Bern University Hospital and University of Bern, 3010 Bern, Switzerland; Translational Imaging Center (TIC), Swiss Institute for Translational and Entrepreneurial Medicine, 3010 Bern, Switzerland; Department of Psychiatry and Psychotherapy, Medical University of Vienna, 1090 Vienna, Austria; Department of Diabetes, Endocrinology, Nutritional Medicine and Metabolism, Inselspital, Bern University Hospital and University of Bern, 3010 Bern, Switzerland; Department of Diabetes, Endocrinology, Nutritional Medicine and Metabolism, Inselspital, Bern University Hospital and University of Bern, 3010 Bern, Switzerland; Translational Imaging Center (TIC), Swiss Institute for Translational and Entrepreneurial Medicine, 3010 Bern, Switzerland; Division of Neuropaediatrics, Development and Rehabilitation, Department of Paediatrics, Inselspital, Bern University Hospital and University of Bern, 3010 Bern, Switzerland

**Keywords:** phenylketonuria, diffusion tensor imaging, ^1^H spectroscopy, cerebral white matter, cognition

## Abstract

Despite increasing knowledge about the effects of phenylketonuria on brain structure and function, it is uncertain whether white matter microstructure is affected and if it is linked to patients’ metabolic control or cognitive performance. Thus, we quantitatively assessed white matter characteristics in adults with phenylketonuria and assessed their relationship to concurrent brain and blood phenylalanine levels, historical metabolic control and cognitive performance. Diffusion tensor imaging and ^1^H spectroscopy were performed in 30 adults with early-treated classical phenylketonuria (median age 35.5 years) and 54 healthy controls (median age 29.3 years). Fractional anisotropy and mean, axial and radial diffusivity were investigated using tract-based spatial statistics, and white matter lesion load was evaluated. Brain phenylalanine levels were measured with ^1^H spectroscopy whereas concurrent plasma phenylalanine levels were assessed after an overnight fast. Retrospective phenylalanine levels were collected to estimate historical metabolic control, and a neuropsychological evaluation assessed the performance in executive functions, attention and processing speed. Widespread reductions in mean diffusivity, axial diffusivity and fractional anisotropy occurred in patients compared to controls. Mean diffusivity and axial diffusivity were decreased in several white matter tracts and were most restricted in the optic radiation (effect size *r*_rb_ = 0.66 to 0.78, *P* < 0.001) and posterior corona radiata (*r*_rb_ = 0.83 to 0.90, *P* < 0.001). Lower fractional anisotropy was found in the optic radiation and posterior corona radiata (*r*_rb_ = 0.43 to 0.49, *P* < 0.001). White matter microstructure in patients was significantly associated with cognition. Specifically, inhibition was related to axial diffusivity in the external capsule (*r*_s_ = −0.69, *P* < 0.001) and the superior (*r*_s_ = −0.58, *P* < 0.001) and inferior longitudinal fasciculi (*r*_s_ = −0.60, *P* < 0.001). Cognitive flexibility was associated with mean diffusivity of the posterior limb of the internal capsule (*r*_s_ = −0.62, *P* < 0.001), and divided attention correlated with fractional anisotropy of the external capsule (*r*_s_ = −0.61, *P* < 0.001). Neither concurrent nor historical metabolic control was significantly associated with white matter microstructure. White matter lesions were present in 29 out of 30 patients (96.7%), most often in the parietal and occipital lobes. However, total white matter lesion load scores were unrelated to patients’ cognitive performance and metabolic control. In conclusion, our findings demonstrate that white matter alterations in early-treated phenylketonuria persist into adulthood, are most prominent in the posterior white matter and are likely to be driven by axonal damage. Furthermore, diffusion tensor imaging metrics in adults with phenylketonuria were related to performance in attention and executive functions.

## Teaser

Muri *et al*. show that white matter alterations in early-treated phenylketonuria persist into adulthood and that reductions in diffusion tensor imaging metrics are associated with lower cognitive performance.

## Graphical Abstract

**Graphical Abstract:**
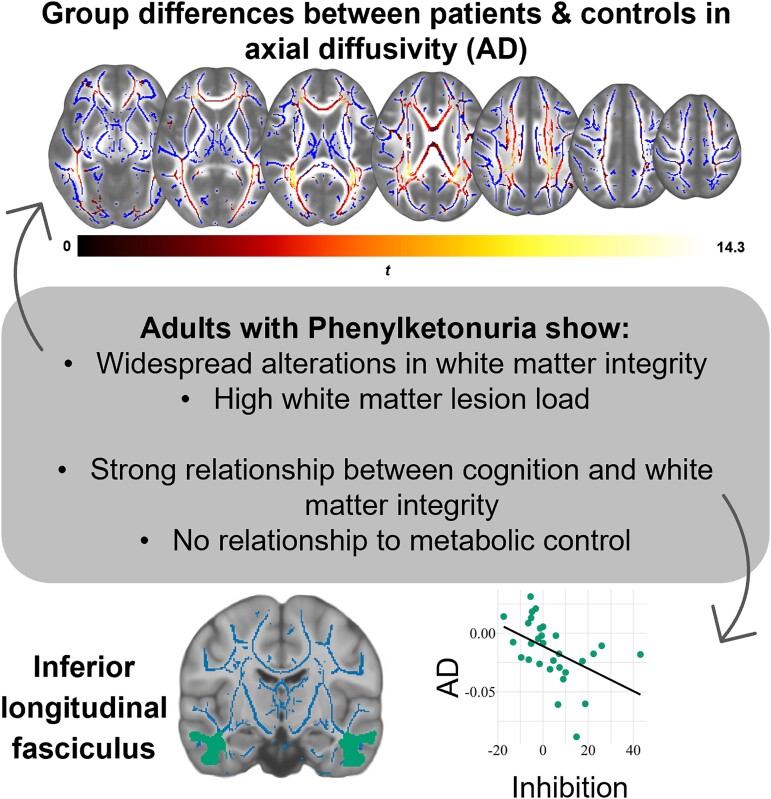

## Introduction

Phenylketonuria (PKU) is a rare inherited metabolic disorder associated with a deficiency in the enzyme phenylalanine (Phe) hydroxylase. This defect prevents the metabolization of Phe and results in high Phe levels in blood and brain. If untreated during childhood, PKU leads to severe, irreversible cognitive impairments and neurological abnormalities. A low-protein diet combined with Phe-free amino acid supplementation, initiated soon after birth, can effectively prevent the development of these impairments. However, despite early detection of PKU and management of the dietary Phe intake during childhood and adolescence, studies indicate that adult patients still exhibit slight cognitive abnormalities when analysed on a group level. Most prominently, general intelligence, executive functions and attention are altered in adults with PKU,^1^ although they are often still within the normative range.^2^

Altered white matter (WM) in PKU is one of the findings most consistently reported in the literature. Periventricular WM hyperintensities on T_2_-weighted images are recognised as the neuroimaging hallmarks of PKU and are found in most patients regardless of their current treatment status.^3–5^ In recent decades, diffusion tensor imaging (DTI) has emerged as a technique to investigate microstructural changes in WM tracts. DTI studies showed marked differences between paediatric patients with PKU and healthy controls. Among the WM structures particularly affected in children and adolescents, or mixed-age samples, were commissural, projection and association fibres such as the corpus callosum,^6–10^ corona radiata,^7,8^ and the superior longitudinal fasciculus,^7,8^ respectively. Other compromised association fibres included the external capsule,^8^ the optic radiation^6^ and the sagittal stratum.^8^ With respect to the projection fibres, the internal capsule has also been reported to be affected in PKU.^7^ Despite efforts to study PKU-related WM abnormalities across different age ranges, it is still unclear whether WM alterations persist into adulthood. To date, two studies on small samples of adult patients suggest that the WM microstructure might also be compromised in adults with PKU (Vermathen *et al*.,^9^*n* = 9 patients; Ding *et al*.,^11^*n* = 4 patients). However, due to the small sample sizes, the robustness and interpretability of the results remain uncertain, and generalisability is limited.

Attempts have been made to investigate brain–behaviour relationships by linking paediatric patients’ cognitive abilities with WM microstructure (e.g. Antenor-Dorsey *et al*.^6^). However, performance in attention and other subdomains of executive functioning, such as inhibition and cognitive flexibility, has so far not been investigated. Furthermore, the relationship between cognitive performance and WM microstructure has yet to be examined in adults with PKU.

For clinical and research purposes, understanding the interplay between metabolic control and WM alterations in adults with PKU is equally important with respect to children. Vermathen *et al*.^9^ found a correlation between concurrent blood and brain Phe levels and mean diffusivity in WM lesions and the corpus callosum in nine adult patients with PKU. Whether such a relationship exists with other WM tracts and historical metabolic control in adult patients with PKU has not yet been explored.

The goal of the current study was to investigate WM characteristics in a relatively large (*n* = 30), sample of adults with early-treated classical PKU compared to healthy controls (*n* = 54). Moreover, we aimed to assess the association between WM characteristics, cognitive performance and metabolic control in patients with PKU. Specifically, we hypothesised that DTI metrics [fractional anisotropy (FA), mean, axial and radial diffusivity (MD, AD and RD)] and total WM lesion load (i) significantly differ between patients with PKU and healthy controls, (ii) are correlated with performance in tasks assessing processing speed, attention and executive functions in patients and (iii) are associated with patients’ concurrent blood and brain Phe levels as well as their historical metabolic control.

## Materials and methods

### Participants

A total of 30 early-treated adults with classical PKU and 55 demographically comparable healthy control participants were recruited within the framework of the Phenylalanine and its Impact on COgnition study (PICO) study (clinicaltrials.gov registration number: NCT03788343)^12^ between July 2019 and April 2022. Patients with PKU (*n* = 30, 13 females, median age = 35.5 years, interquartile range (IQR) = 12.3, age range = 19–48 years) were recruited through their metabolic specialists at the university hospitals of Bern, Zurich, Lausanne and Basel and the Cantonal Hospital St. Gallen (Switzerland), the university hospitals of Ulm and Hamburg (Germany) and Innsbruck (Austria). Patients were included if they were 18 years or older, had had a positive newborn screening test for PKU and were treated with a Phe-restricted diet within the first 30 days of life. Patients were excluded if Phe concentrations 6 months before study participation exceeded 1600 μmol/L, they had an untreated vitamin B12 deficiency or were pregnant, breastfeeding or unwilling to use a highly efficient contraception method during study participation.

Control participants aged 18 years or older were recruited in and around Bern and Zurich through advertisements and word-of-mouth. One control participant was excluded due to an incidental MRI finding that biased the results of the DTI analysis, leaving a total of 54 control participants (26 females, median age = 29.3 years, IQR = 9.4, age range = 18–53 years). The demographical variables age, sex, education and IQ of the included patients and controls are further described in Supplementary Table 1.

Exclusion criteria for patients and controls included: a history of neurological disorders, severe psychiatric conditions or any contraindication to MRI (e.g. metal implants). All participants provided written informed consent before study participation. The study was performed according to the Declaration of Helsinki and was approved by the Cantonal Ethics Committee Bern, Switzerland (2018-01609).

### Neuroimaging

MRI was performed after an 8–12-hour overnight fast. MRI and ^1^H spectroscopy data of all 84 participants were acquired on a 3T Siemens Prisma MRI scanner at the Translational Imaging Center (TIC) within the Swiss Institute for Translational and Entrepreneurial Medicine (sitem-insel), Bern, Switzerland. The scanner was equipped with a 64-channel head coil with an integrated mirror allowing participants to watch a tranquil nature documentary during structural image acquisition.

### MRI acquisition

For all participants, a high-resolution (1 mm^3^) T_1_-weighted image (MPRAGE) was collected (repetition time TR = 1950 ms, echo time TE = 2.26 ms, inversion time TI = 900 ms, acquisition time TA = 4:34 min, flip angle = 9, in-plane resolution = 1 × 1 mm, slice thickness = 1 mm, number of slices = 176, field of view = 256 mm × 256 mm, matrix = 256 × 256).

T_2_-weighted images were also obtained (axial: 0.5 × 0.5 × 3.0 mm, TR = 4800 ms, TE = 88 ms, TA = 1:04 min; sagittal: 1.0 × 1.0 × 4.0 mm, TR = 3000 ms, TE = 84 ms, TA = 0:26 min; and coronal: 1.0 × 1.0 × 4.0 mm, TR = 3000 ms, TE = 84 ms, TA = 0:23 min).

Diffusion-weighted images were acquired with a spin-echo echo planar imaging sequence using 122 non-collinear directions preceded by a *b* = 0 reference volume (TR = 3700 ms, TE = 87 ms, TA = 7:55 min, slice thickness = 2.2 mm (isotropic), number of slices = 56, phase encoding direction = anterior–posterior, acceleration factor = 2, *q*-space weightings = 3, *q*-space max. *b*-value = 3000 s/mm^2^, full *q*-space coverage).

### ^1^H spectroscopy acquisition

MR spectra were acquired using a semi-LASER sequence^13^ with the manufacturer’s second order shimming routine (option ‘brain’). The transmit voltage was optimised for maximum signal and the variable power and optimised relaxation delays (VAPOR) water suppression (bandwidth of 135 Hz) for minimal residual water signal using semi-automatic acquisition loops for each subject. A large volume of interest of 50 × 75 × 20 mm^3^ (or reduced to 50 × 65 × 20 mm^3^ depending on head geometry) was semi-automatically placed in supraventricular white and grey matters with a small preponderance of WM (∼5 mm spacing to the roof of the lateral ventricles).^14^ TE was set to 35 ms and TR to 2500 ms. The transmit frequency was set to 7.3 ppm for water-suppressed spectra; 256 acquisitions were averaged from two batches of 128 scans (12 min total scan time). In addition, unsuppressed reference scans of the water signal were recorded for each subject for eddy current and phase correction. For water referencing and evaluation of CSF signal contributions, an additional series of eight scans with different TEs (35, 50, 75, 100, 140, 200, 400 and 1000 ms) was recorded with a TR of 6000 ms.

### DTI analysis

#### DTI preprocessing

DTI preprocessing was performed using the FSL toolbox from the Oxford Centre for Functional MRI of the Brain (FMRIB) software library (version 6.0.5).^15^ We followed the processing pipeline suggested by Maximov *et al*.^16^ In short, this included noise corrections^17^ and Gibbs ringing corrections,^18^ as well as eddy current and motion corrections (FSL eddy). The resulting images were skull-stripped using FSL-BET. Bias field corrections^19^ using the Advanced Normalization Tools (ANTs) and spatial smoothing (FSL fslmaths) were applied to finalise the image preprocessing. To fit the diffusion tensor model at each voxel, DTIFIT was applied to the preprocessed images.

#### Tract-based spatial statistics: voxel-wise analysis

We applied tract-based spatial statistics (TBSS) to examine group differences in DTI metrics in an explorative way.^15^ This method generates a WM skeleton on the FA maps and projects FA, MD, AD and RD onto the skeleton. Group differences in each DTI metric were estimated using ‘randomise’ implemented in FSL with 5000 permutations, and threshold-free cluster enhancement with family-wise error was used to correct for multiple comparisons. Finally, *P*-values were corrected for further DTI metric multiplicity (four metrics) using the Bonferroni method. All relevant indices from the whole-brain group analysis (cluster size, MNI coordinates (centre of gravity), maximum *t*-value and *P*-value) were extracted and are reported in Supplementary Table 2. Only clusters that exceed 50 voxels per region were included. Furthermore, we also extracted global FA, MD and AD for each participant to exploratively evaluate their relationship to a general measure of overall cognitive abilities (IQ).

#### Tract-based spatial statistics: region-of-interest analysis

To allow for a better comparability with prior studies, we further analysed WM tracts that have previously been reported to show alterations in paediatric or mixed-aged samples of patients with PKU using a region of interest (ROI) approach. We extracted WM tracts from the Johns Hopkins University (JHU) ICBM-DTI-81 atlas^20,21^ and the XTRACT HCP Probabilistic Tract atlas.^22^ The ROIs previously shown to differ between paediatric and mixed-age samples of patients with PKU and controls included the genu, body and splenium of the corpus callosum, as well as optic radiation, anterior, superior and posterior corona radiata, inferior longitudinal fasciculus, superior longitudinal fasciculus, anterior limb of the internal capsule, posterior limb of the internal capsule and the external capsule.^6–10^ Altogether, 12 main WM tracts were selected for the ROI analysis. To decrease the number of comparisons, we combined the left hemisphere and the right hemisphere for each tract. Only DTI metrics that showed significant differences in the voxel-wise analysis were included in the ROI analysis.

### White matter lesion load

WM lesions on anonymised, and randomised T_2_-weighted images of patients and controls were rated by two board-certified neuroradiologists (P.R. and K.P.), who were blinded to the medical history, age and sex of the subjects. WM abnormalities attributed to PKU were defined as high signal on T_2_-weighted images, which is assumed to reflect intramyelinic oedema in early-treated patients.^23^ Lesions were rated on a scale from 0 to 12, as suggested by Pietz *et al*.^4^ In short, frontal, parietal, temporal and occipital lobes were each rated on a 3-point scale: 0 points indicated no WM involvement, 1 point was given if deep WM was affected and 2 points if images showed subcortical WM involvement. Additionally, brainstem and cerebellum were each rated on a 2-point scale, with 0 points for no involvement and 2 points indicating WM involvement.

### ^1^H spectroscopy analysis

Data processing, including frequency alignment, eddy current correction, signal averaging and residual water signal removal with a Hankel Lanczos singular value decomposition filter, was performed in jMRUI.^24^ Phe was quantified as described by Kreis *et al*.^25^ The spectra were scaled by the size of the parenchymal water signal from a biexponential fit of the TE-series (thus compensating for CSF water signal contributions).

Spectra were fitted in FiTAID^26^ for both the upfield (20 metabolites and a macromolecular background spectrum) and downfield parts. The fitting model for the largely ill-defined downfield part of the spectrum was based on summed spectra from healthy subjects and from patients with PKU. Besides the simulated responses of Phe, homocarnosine (hCs) and *n*-acetylaspartate (NAA), 20 Voigt lines (with heuristically optimised frequencies as well as Lorentzian and Gaussian broadening) were included. For the per subject fits, it was assumed that the relative composition of the heuristic background signals does not change between subjects (verified by inspection of difference signals and fit residuals), and only the amplitudes of the known metabolites NAA, hCs and Phe plus the overall amplitude of the background signals were adapted. The Phe content was quantified based on an assumed water content of the parenchymal signal estimated from literature recommendations^27^ and white/grey matter content assumptions for the ROI. Lacking published values for 3T, T_1_ and T_2_ relaxation effects were considered assuming relaxation constants of 890 and 124 ms for T_1_ and T_2_, respectively, comparable to mean values of the methylene signal of creatine for white and grey matters.^28^

### Metabolic control

#### Concurrent metabolic parameters

Blood sampling was performed after an 8–12-hour overnight fast and before the MRI examination to determine plasma Phe, tyrosine (Tyr) and tryptophan (Trp) concentrations. High-performance ion-exchange liquid chromatography with post-column photometric detection of ninhydrin-derivatised amino acids was applied.

#### Historical metabolic parameters

Historical metabolic control was estimated using the index of dietary control (IDC), which is calculated as the mean of the yearly medians of all available Phe levels measured throughout the patients’ life (for more details, see Muri *et al*.^29^). Four age categories were created corresponding to distinct developmental periods: 0–5 years, 6–12 years, 13–17 years and 18+ years. Each patient had to have a minimum of 10 measurements per age category to calculate the IDC (see also Weglage *et al*.^30^). Additionally, we computed lifetime Phe concentration. Descriptive statistics for these parameters have been published by Muri *et al*.^29^

### Cognitive assessment

The cognitive assessment was performed according to the PICO study protocol.^12^ The Wechsler Adult Intelligence Scale Fourth Edition (WAIS-IV)^31^ was used to evaluate general intelligence (IQ), with the subtests Matrix Reasoning, Vocabulary, Arithmetic and Symbol Search being administered. Assessment of executive functions was based on the model of Miyake *et al*.^32^ and included working memory [*n*-back task of the Test of Attentional Performance (TAP)],^33^ inhibition and cognitive flexibility (colour–word interference test conditions 3 and 4, respectively) [Delis–Kaplan Executive Function System (D-KEFS)].^34^ Attention was evaluated using the TAP subtests Alertness, Divided Attention and Sustained Attention.^32^ These cognitive tasks and the corresponding performance measures (accuracy and reaction time) are further described in Muri *et al*.^29^ Processing speed was calculated as the total correct responses in the subtest Symbol Search of the WAIS-IV.

### Statistical analysis

The data were analysed with R version 4.1.2.^35^ As most continuous variables showed non-normal distribution, medians and IQR were reported for all variables. The blood–brain ratio of Phe was calculated by dividing concurrent blood Phe levels (μmol/L) by brain Phe levels measured by ^1^H spectroscopy (mmol/kg was converted into μmol/L). Voxel-wise, global DTI and ROI-based TBSS analyses were corrected for age and sex to take into account evidence for age and sex differences in DTI metrics.^36–38^ As for the tracts extracted with the ICBM-DTI-81 and XTRACT atlases, linear regression models were built with raw DTI metrics as the dependent variable and age and sex as independent variables. Residuals for FA, MD and AD for each ROI were calculated for the patient group based on the control group. Likewise, residuals from linear regression were calculated for all raw cognitive variables with age as a covariate. Raw residuals for DTI and cognition were used for further group and correlation analyses. Differences between patients and controls regarding age, DTI metrics (residuals), total WM lesion scores and cognitive performance (residuals) were examined with Mann–Whitney U tests and rank-biserial correlation coefficients (*r*_rb_) as corresponding effect sizes. Differences in DTI metrics of the optic radiation between patients with and without WM lesion load in the occipital lobe were analysed with Mann–Whitney U tests. Spearman’s rank correlation coefficients (*r*_s_) were used to investigate the relationship between cognitive performance (residuals), DTI metrics (residuals) and metabolic variables. Bootstrapping (*n* = 1000) was used to calculate confidence intervals for Spearman correlation. *P*-values of <0.05 were considered significant and are reported as uncorrected, two-sided *P*-values, for which we further specified whether they survived correction for multiple comparisons using the false discovery rate (FDR).^39^*P*-values for each hypothesis were FDR corrected. Effect sizes were interpreted as suggested by Funder and Ozer:^40^ very small *r* ≥ 0.05, small *r* ≥ 0.10, medium *r* ≥ 0.20, large *r* ≥ 0.30 and very large *r* ≥ 0.40.

## Results

### WM microstructure

**Figure 1:**
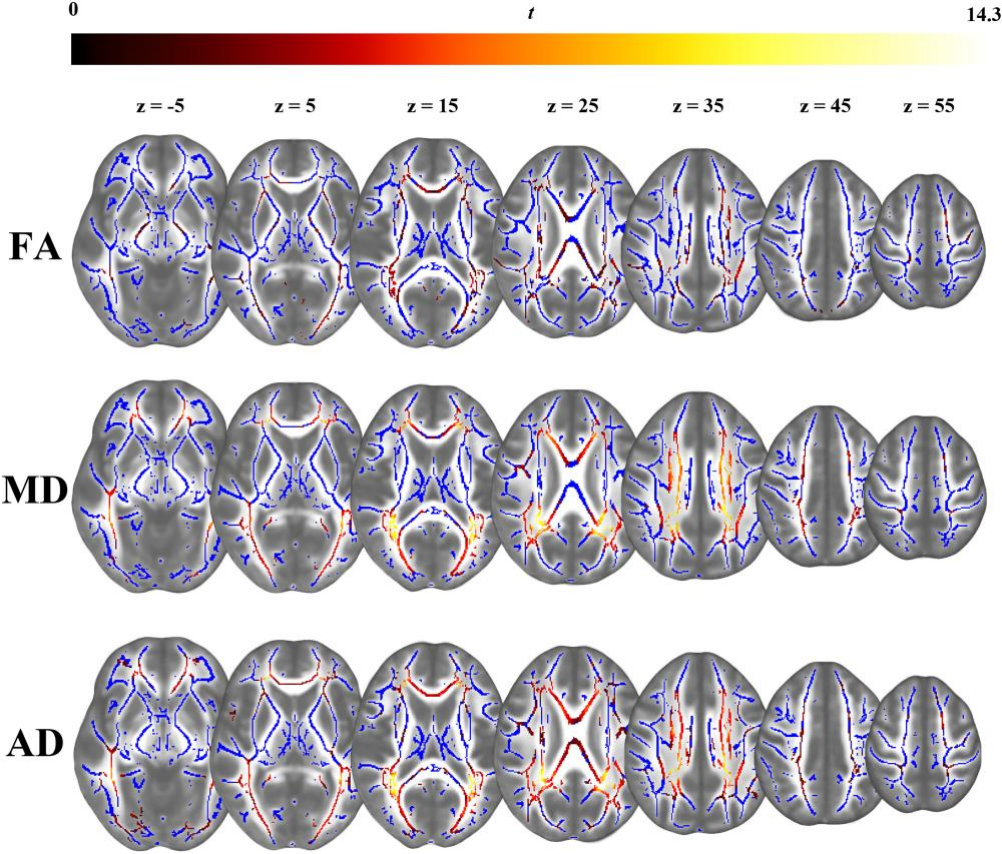
**Group differences in WM microstructure.** Differences in WM microstructure between patients and controls as shown by tract-based spatial statistics (TBSS), corrected for multiple comparisons (*P* < 0.05). Blue regions represent the mean FA skeleton. Voxel-wise differences are marked on a colour continuum (i.e. the lighter the lower in patients) and displayed for fractional anisotropy (FA, upper row), mean diffusivity (MD, middle row), and axial diffusivity (AD, bottom row). Radial diffusivity (RD) did not differ significantly between the two groups and is therefore not shown here

Patients with PKU showed lower global FA [*r*_rb_ = 0.34, *P* = 0.012, 95% CI (0.09, 0.55)], and lower global AD [*r*_rb_ = 0.33, *P* = 0.014, 95% CI (0.08, 0.54)] than controls but no group difference was seen in global MD. Whole-brain TBSS analysis revealed that patients with PKU had significantly lower FA, MD and AD across the brain than controls (Fig. 1). RD did not significantly differ between patients and healthy controls. Clusters of significant group differences in WM integrity are reported in Supplementary Table 2.

Detailed results of the ROI analysis are displayed in Table 1. Inspection of the effect sizes in the ROI analysis revealed that MD and AD were greatly decreased in the optic radiation and the posterior corona radiata. Furthermore, significant reductions of the MD and AD were seen in the genu, body and splenium of the corpus callosum, superior longitudinal fasciculus, and anterior and superior corona radiata. Likewise, FA in patients was significantly lower in the optic radiation and the posterior corona radiata compared to controls. In the inferior longitudinal fasciculus, AD, but no other DTI metric, was lower in patients than in controls.

**Table 1:** Group differences in DTI metrics for all ROIs

ROIs	Fractional anisotropy (FA)				Mean diffusivity (MD)				Axial diffusivity (AD)			
	PAT	CON	*P*	ES^d^	PAT	CON	*P*	ES^d^	PAT	CON	*P*	ES^d^
G-CC^a^	0.76 0.03	0.76 0.02	0.230	0.16 [−0.10, 0.40]	**0.43** **0.02**	**0.44** **0.02**	**0**.**011**	**0.34** **[0.09, 0.54]**	**0.92** **0.05**	**0.95** **0.04**	**0**.**012**	**0.33** **[0.09, 0.54]**
B-CC^a^	0.75 0.02	0.77 0.02	0.039	0.27 [0.02, 0.49]	**0.43** **0.02**	**0.45** **0.02**	**<0**.**001**	**0.55** **[0.35, 0.71]**	**0.92** **0.06**	**0.96** **0.04**	**<0**.**001**	**0.62** **[0.44, 0.76]**
S-CC^a^	0.83 0.02	0.83 0.02	0.077	0.23 [−0.02, 0.46]	**0.40** **0.02**	**0.41** **0.01**	**0**.**002**	**0.42** **[0.18, 0.61]**	**0.93** **0.03**	**0.96** **0.04**	**<0**.**001**	**0.47** **[0.25, 0.65]**
EC^b^	0.48 0.02	0.49 0.03	0.245	0.15 [−0.10, 0.39]	0.49 0.02	0.49 0.02	0.805	−0.03 [−0.28, 0.22]	0.77 0.02	0.77 0.03	0.929	0.01 [−0.24, 0.27]
ILF^b^	0.42 0.02	0.43 0.02	0.037	0.28 [0.02, 0.50]	0.49 0.01	0.49 0.02	0.213	0.17 [−0.09, 0.40]	**0.72** **0.02**	**0.74** **0.03**	**0**.**007**	**0.36** **[0.12, 0.56]**
SLF^b^	0.57 0.03	0.59 0.03	0.061	0.25 [−0.01, 0.47]	**0.41** **0.02**	**0.42** **0.02**	**0**.**005**	**0.37** **[0.13, 0.57]**	**0.71** **0.04**	**0.73** **0.03**	**<0**.**001**	**0.48** **[0.26, 0.66]**
OR^b^	**0.56** **0.02**	**0.58** **0.02**	**<0**.**001**	**0.49** **[0.27, 0.66]**	**0.44** **0.02**	**0.46** **0.02**	**<0**.**001**	**0.66** **[0.49, 0.78]**	**0.76** **0.06**	**0.81** **0.03**	**<0**.**001**	**0.78** **[0.65, 0.86]**
ACR^c^	0.52 0.05	0.54 0.04	0.045	0.27 [0.01, 0.49]	**0.41** **0.01**	**0.43** **0.02**	**<0**.**001**	**0.54** **[0.34, 0.70]**	**0.68** **0.04**	**0.72** **0.04**	**<0**.**001**	**0.55** **[0.34, 0.70]**
SCR^c^	0.56 0.03	0.57 0.03	0.087	0.23 [−0.03, 0.45]	**0.39** **0.01**	**0.40** **0.02**	**<0**.**001**	**0.50** **[0.29, 0.67]**	**0.66** **0.04**	**0.69** **0.03**	**<0**.**001**	**0.53** **[0.32, 0.69]**
PCR^c^	**0.54** **0.04**	**0.55** **0.02**	**0**.**001**	**0.43** **[0.20, 0.62]**	**0.40** **0.03**	**0.44** **0.02**	**<0**.**001**	**0.83** **[0.74, 0.90]**	**0.66** **0.06**	**0.73** **0.03**	**<0**.**001**	**0.90** **[0.83, 0.94]**
ALIC^c^	0.63 0.02	0.64 0.03	0.063	0.25 [−0.01, 0.47]	0.42 0.02	0.41 0.01	0.160	−0.19 [−0.42, 0.07]	0.76 0.02	0.76 0.02	0.585	−0.07 [−0.32, 0.18]
PLIC^c^	0.76 0.03	0.77 0.03	0.094	0.22 [−0.03, 0.45]	0.39 0.01	0.39 0.02	0.631	0.06 [−0.19, 0.31]	0.82 0.02	0.83 0.04	0.139	0.20 [−0.06, 0.43]

Data are presented as age- and sex-corrected median DTI metrics with interquartile ranges. MD and AD are displayed as mm^2^/s × 10^−3^. Results that survive FDR correction are marked in bold.

ACR, anterior corona radiata; ALIC, anterior limb of the internal capsule; B-CC, body of the corpus callosum; CON, controls; EC, external capsule; ES, effect size; G-CC, genu of the corpus callosum; ILF, inferior longitudinal fasciculus; *P*, *P*-value; PAT, patients; OR, optic radiation; PCR, posterior corona radiata; PLIC, posterior limb of the internal capsule; ROIs, regions of interest; S-CC, splenium of the corpus callosum; SCR, superior corona radiata; SLF, superior longitudinal fasciculus.

a Commissural fibres.

b Association fibres.

c Projection fibres.

d Effect sizes are reported as rank-biserial correlation coefficients (*r*_rb_) for Mann–Whitney U tests with the corresponding 95% confidence interval in square brackets.

### WM lesion load

WM lesions were found in 29 out of 30 patients (96.7%) and two out of 54 controls (3.7%). Total WM lesion scores ranged from 0 to 7 (median = 2.5, IQR = 1.75) in patients and from 0 to 1 (median = 0.0, IQR = 0.00) in controls, revealing significant differences between the two groups [*r*_rb_ = −0.96, *P* < 0.001, 95% CI (−0.97, −0.93)]. In patients, the parietal (*n* = 29, 96.7%) and occipital lobes (*n* = 19, 63.3%) were most frequently affected, followed by the frontal lobe (*n* = 15, 50.0%), temporal lobe (*n* = 4, 13.3%), brainstem (*n* = 3, 10.0%) and cerebellum (*n* = 2, 6.7%). There was no significant difference in DTI metrics (FA, MD and AD) of the optic radiation between patients with and without WM lesion load in the occipital lobe. The total WM lesion score was not correlated with cognitive performance, concurrent blood Phe, concurrent brain Phe or historical Phe levels.

### Concurrent and historical metabolic control

The median Phe level of 741 μmol/L (IQR = 358, min = 380, max = 1208) in patients was above the recommended level of 600 μmol/L proposed by the current European guidelines.^41^ On an individual level, eight patients (26.7%) had concentrations below 600 μmol/L. Median Tyr was 38 μmol/L (IQR = 12, min = 28, max = 71), and median Trp level was 38 μmol/L (IQR = 10, min = 21, max = 54).

**Figure 2:**
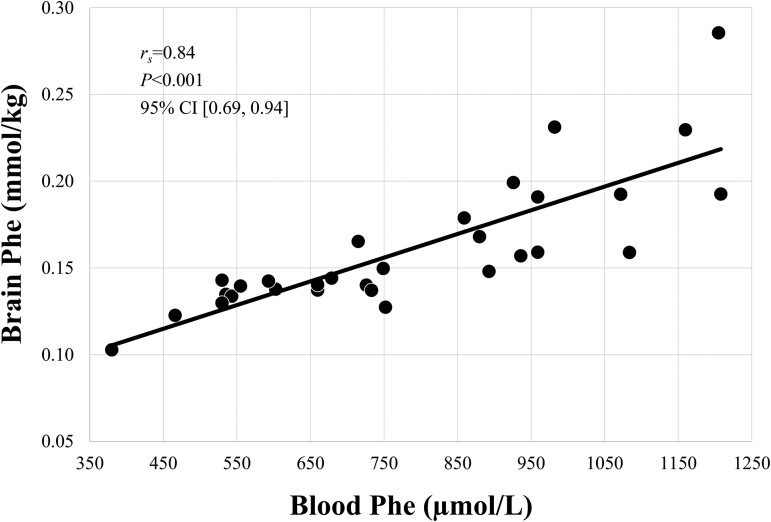
**Relationship between brain and blood Phe.** Correlation between brain Phe levels as measured by ^1^H spectroscopy and blood Phe levels in *n* = 30 patients with PKU. CI, confidence interval; *r*_s_, Spearman’s rank correlation coefficient; *P*, *P*-value; Phe, phenylalanine

In patients, brain Phe levels were highly correlated with levels in blood [*r*_s_ = 0.84, *P* < 0.001, 95% CI (0.69, 0.94)] (Fig. 2) but were unrelated to blood Tyr levels [*r*_s_ = 0.03, *P* = 0.865, 95% CI (−0.34, 0.41)] and Trp levels [*r*_s_ = −0.16, *P* = 0.394, 95% CI (−0.54, 0.21)]. The median of the blood–brain ratio was 4.53 (IQR = 1.09, min = 3.52, max = 6.50).

### Cognitive performance and metabolic control

Patients performed significantly worse than controls in tasks that measured working memory [*r*_rb_ = −0.40, *P* = 0.003, 95% CI (−0.59, −0.16)], cognitive flexibility [*r*_rb_ = 0.45, *P* = 0.001, 95% CI (0.22, 0.63)], sustained attention [*r*_rb_ = 0.40, *P* = 0.003, 95% CI (0.17, 0.60)] and processing speed [*r*_rb_ = −0.41, *P* = 0.002, 95% CI (−0.17, −0.60)] (see also Supplementary Table 3). IDC lifetime was significantly related to performance in the task assessing processing speed [*r*_s_ = 0.55, *P* = 0.044, 95% CI (0.00, 0.85)], but this correlation did not survive FDR correction. None of the other concurrent or historical metabolic parameters was significantly correlated with patients’ cognitive performance. Some of the results on cognitive performance and its correlation to metabolic control have already been published.^29^

The eight patients whose baseline Phe levels were below the recommended Phe levels (600 μmol/L) were compared to the 22 patients with Phe levels above the recommended Phe levels regarding cognitive performance. There was no significant difference between the patients above and below the guidelines in any of the cognitive tasks.

### Relationship between WM, cognitive performance and metabolic control

FA and MD of various ROIs were significantly correlated with concurrent and historical metabolic control (see Supplementary Table 4). However, none of these correlations survived FDR correction.

**Figure 3:**
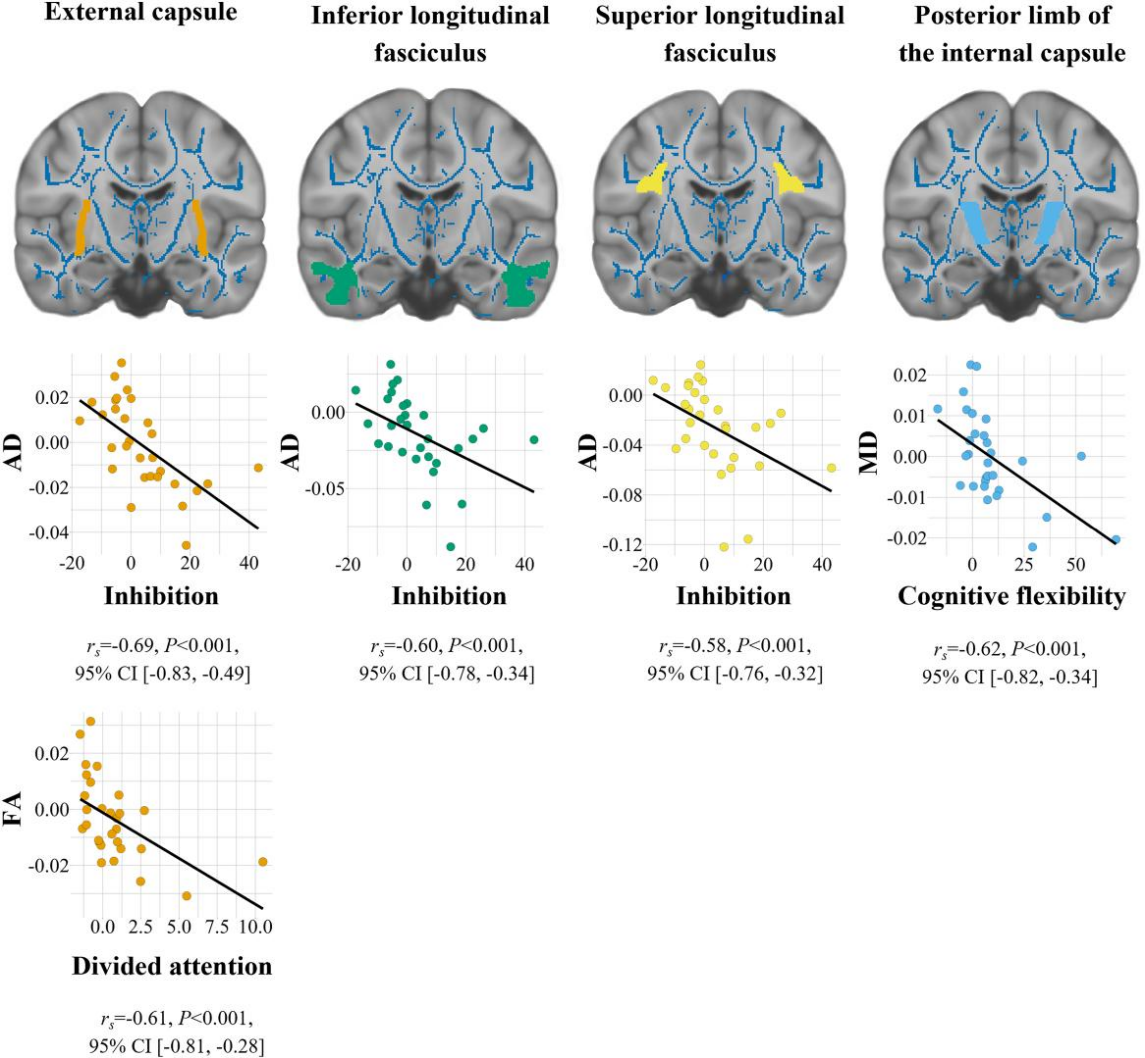
**Relationship between WM and cognition.** Significant Spearman’s rank correlations between DTI metrics within regions of interest (as marked in the brain images) and cognitive performance in *n* = 30 patients with PKU (*n* = 29: correlation between FA of the external capsule and divided attention). The results remained significant after removal of outliers and after controlling for IQ. Residual scores for AD and MD are displayed as mm^2^/s × 10^−3^. The higher the residual scores for cognition, the lower the performance in the respective task. CI, confidence interval; *r*_s_, Spearman’s rank correlation coefficient; *P*, *P*-value

In patients, IQ was unrelated to global FA, MD and AD. Performance in tasks assessing inhibition was significantly correlated with AD in the external capsule and the superior and inferior longitudinal fasciculi (Fig. 3). Cognitive flexibility was associated with MD of the posterior limb of the internal capsule, and divided attention correlated with FA of the external capsule (Fig. 3). All these correlations survived FDR correction. The results remained significant after removal of outliers and after controlling for IQ. Significant correlations that did not survive FDR correction, including processing speed, inhibition, divided attention and alertness, are presented in Supplementary Table 5.

Compared to patients above 600 μmol/L, patients below 600 μmol/L showed significantly lower FA in the splenium of the corpus callosum [*r*_rb_ = −0.56, *P* = 0.021, 95% CI (−0.80, −0.15)], higher MD in the external capsule [*r*_rb_ = 0.52, *P* = 0.031, 95% CI (0.10, 0.78)] and higher AD in the anterior limb of the internal capsule [*r*_rb_ = 0.52, *P* = 0.031, 95% CI (0.10, 0.78)]. These group difference did not survive FDR correction.

In controls, IQ was unrelated to global FA, MD and AD. There were some significant correlations between cognitive performance and DTI metrics with medium to very large effects, which did not survive FDR correction (Supplementary Table 6).

## Discussion

In a sample of 30 adults with early-treated classical PKU, we found global and regional WM microstructural abnormalities. The most prominent reductions were in MD and AD but were also observed in FA, and the largest effect sizes were in the posterior WM (*r*_rb_ = 0.66 to 0.90). Twenty-nine patients showed WM lesions on T_2_-weighted imaging, with the highest lesion scores in the parietal and occipital lobes. In patients, lower performance in tasks assessing inhibition, cognitive flexibility and divided attention was correlated with lower DTI metrics (*r*_s_ = −0.58 to −0.69). However, neither concurrent blood and brain Phe nor historical metabolic control was significantly correlated with DTI metrics.

The optic radiation and posterior corona radiata—both tracts located in the posterior part of the brain—showed the largest decreases in DTI metrics (MD and AD) in patients. Using a more localised DTI approach, Clocksin *et al*.^42^ presented results consistent with ours suggesting that WM alterations are most evident in posterior WM tracts in a mixed-age sample of patients with PKU. These findings are in agreement with our earlier observations on cortical grey matter in the same sample of patients, showing the most pronounced cortical thickness alterations in posterior brain regions.^29^ The optic radiation is one of the first tracts to reach myelin maturity in the developing brain.^43^ Myelination, in general, occurs in early postnatal life, with the fastest myelination rates in the first 8 months after birth.^43,44^ The developmental trajectory of the WM demonstrates why early-initiated and continuous treatment of PKU in childhood is vital for normal brain development.

The largest effects were found for AD in the optic radiation and posterior corona radiata, although AD reductions also involved the anterior and superior corona radiata, corpus callosum and the superior and inferior longitudinal fasciculi. In a mixed sample of paediatric and adult patients with PKU, Peng *et al.*^8^ also reported lower AD in the corpus callosum, superior longitudinal fasciculus and corona radiata. Decreased AD is thought to indicate axonal damage,^45^ whereby the disintegration of axons may result in barriers that hinder the movement of water along the axons, in turn reducing diffusion parallel to the axon fibres.^46^ Since RD was unchanged in our sample, differences in MD between patients and controls are likely to be a consequence of reduced AD. Previous studies on WM microstructural alterations in patients with PKU most frequently investigated differences in MD (for an overview, see González *et al*.^7^), whereas the combination of all four DTI metrics has seldom been explored.^8,47^ Like MD and AD, decreases in FA were also found in the optic radiation and posterior corona radiata. Although our results differ from those of previous studies reporting increases or no changes in FA in paediatric^7,10,47,48^ and mixed-age samples with PKU,^6,8^ our findings are consistent with the results of Vermathen *et al.*^9^ demonstrating decreased FA in nine adults with early-treated PKU. Reductions in FA can be driven by decreases in AD and/or increases in RD. In this context, Vermathen *et al*.^9^ argued that differences in FA in their study mainly resulted from changes in the longitudinal diffusion direction (i.e. driven by AD), which our findings support. The combined finding of reduced AD, MD and FA was previously reported in healthy older adults and was associated with acute axonal damage.^49^

WM hyperintensities on T_2_-weighted images were found in 29 out of 30 adults with PKU aligning with previous studies.^50,51^ These WM abnormalities were most frequently located in the parietal lobes but were also found in the occipital and frontal lobes. Similarly, in a mixed sample of early- and late-treated patients with PKU, WM lesions were most apparent in the parietal lobe, followed by the occipital, frontal and temporal lobes.^50^ The literature suggests that the typical WM hyperintensities in early-treated PKU reflect intramyelinic oedema,^3^ a form of cytotoxic oedema. This assumption is further corroborated by the recent finding that Phe can increase membrane permeability,^52^ likely due to Phe aggregation within the membranes. On a related note, Rondelli *et al*.^53^ propose that Phe affects the normal wrapping of myelin sheaths and interferes with glycosphingolipids-interaction resulting in increased vacuolisation.

Notably, WM alterations revealed by DTI were somehow detached from WM lesion load ratings in patients with PKU. A recent study on patients with Fabry disease, another congenital error affecting the glycosphingolipid metabolism, also demonstrated that WM abnormalities can be detected in normal-appearing WM with DTI.^54^ Therefore, DTI represents a sensitive technique to gain unique information about the WM microstructural integrity in inherited metabolic diseases not apparent on conventional MRI.

Taken together, the combination of lower AD, MD and FA in patients with PKU points to axonal injury, and white matter lesions were not associated with DTI metrics.

Our finding on the high correlation between blood and brain Phe levels aligns with previous reports^9,55^ and further opposes the notion of an interindividual variability of Phe transport across the blood–brain barrier. Moreover, the blood–brain ratio of 4.53 in our study is comparable with that of Rupp *et al*.^55^ and indicates that the Phe concentration in the brain is considerably lower than that in the blood.

We found significant correlations between DTI metrics and concurrent plasma Phe and Tyr, as well as historical metabolic control, with large effect sizes that did not, however, survive FDR correction. The direction of these results aligns with earlier research on paediatric, mixed-age and adult patient samples reporting an association between MD and concurrent Phe^6,7,9,50,56^ and MD and historical Phe levels.^8,57^ Considering the limited sample size and the large effect sizes of our results, the inclusion of a larger number of adults with PKU may have lowered the *P*-value leading to survival of the multiple comparison correction. Furthermore, Mastrangelo *et al*.^58^ demonstrated that the duration of exposure to high Phe levels may impact the progression of white matter alterations. Such a longitudinal investigation may further help to unravel the effects of PKU on the WM. Likewise, our results showed that Phe levels directly measured in the brain with ^1^H spectroscopy were significantly correlated with DTI metrics but, again, did not retain significance after FDR correction. Two previous studies described a significant association between WM and brain Phe level.^9,50^ Both investigated this relationship in localised WM lesions and not in major WM tracts, indicating that high brain Phe levels might have a more localised effect resulting in intramyelinic oedema. Going beyond structural correlates, functional imaging markers may be more sensitive to concurrent metabolic parameters (e.g. Abgottspon *et al*.^61^ and Christ *et al*.^62^). Furthermore, the more widespread WM alterations of major WM tracts found in our study may reflect a more diffuse indirect influence of high Phe levels—expressed as a general disturbance of Phe metabolism, including catecholamine neurotransmitters. In that respect, Kylies *et al*.^59^ suggested that high Phe levels are only a proxy for cerebral damage, as Phe itself might not directly contribute to the clinical manifestations of PKU. These authors gave more weight on another hypothesis, namely that neurotransmitter depletion could be a reason for brain dysfunction in PKU. This hypothesis was further substantiated in a Pah-enu2 mouse model showing strong negative correlations between plasma Phe levels and brain serotonin, dopamine and norepinephrine levels.^60^ Overall, these differing hypotheses reflect the multifactorial nature of PKU that is accompanied by a cascade of metabolic changes affecting brain structure and function.

In our patients, the general measure of IQ was unrelated to global DTI metrics. However, lower DTI metrics in ROIs were linked to poorer performance in specific cognitive subdomains, even after controlling for IQ. Specifically, inhibition was significantly related to AD in the external capsule and the superior and inferior longitudinal fasciculi. All three of these WM tracts contain cortico-cortical association fibres and are crucial in cognitive processes such as attention,^63^ language^64,65^ and executive functions.^66^ WM alterations in the external capsule have previously been linked to executive dysfunction during healthy aging.^66^ In the current study, inhibitory control was assessed with the Stroop paradigm, in which the speed of colour-naming is measured. As a colour–word interference test, it contains a certain language component. The superior longitudinal fasciculus of the ICBM-DTI-81 atlas includes language-related areas,^20^ and the inferior longitudinal fasciculus was found to play a role in language comprehension.^67^ The language component in this task might partly explain the strong correlations between inhibition and the superior and inferior longitudinal fasciculi. The correlations between DTI metrics and cognitive performance were observed in both patients and controls. However, correlation coefficients (*r*-values) were generally higher in patients than in controls (see Supplementary Tables 5 and 6).

Interestingly, the cognitive tasks relating to WM microstructure were not associated with cortical grey matter in the identical patient sample.^29^ This indicates that grey matter alterations described in our previous study might be driven mainly by WM compromise, possibly resulting from sustained oedematous inflammation processes. Together, the results of our previous and present study suggest that cognitive performance might be more closely related to WM than to grey matter abnormalities in adults with PKU. Thus, our findings further substantiate the assumption that the compromised WM in adults with PKU is one possible determinant of the slight cognitive alterations in various subdomains.

It is noteworthy that patients with concurrent Phe levels above (*n* = 22) and below (*n* = 8) the European guidelines differed in some DTI metrics (although the results did not survive FDR correction), while demonstrating comparable cognitive performance. However, the unequal sample sizes between the two groups may limit the interpretation of these findings. Future studies could benefit from recruiting larger samples and examining a wider range of Phe levels to further explore the relationship between PKU and cognitive function.

A few limitations to this study need to be acknowledged. Firstly, FA results must be interpreted with caution as FA can be decreased in regions with complex tissue microstructure (i.e. crossing fibres). This issue could be circumvented in future studies by using more advanced techniques such as high angular resolution diffusion-weighted imaging. Furthermore, although FA is sensitive to change, it is also an inherently nonspecific measure for which it is practically impossible to determine individual microscopic contributions. To disentangle key contributors to FA, future studies could apply a newer approach known as neurite orientation dispersion and density imaging.^68^ Additionally, the broad range of Phe levels observed in our patient sample may have contributed to an increased variability in the clinical characteristics of our sample. Lastly, although TBSS analysis offers higher statistical power than voxel-based analyses and simultaneously allows for an explorative approach, variations in peripheral WM tracts might be missed.

In conclusion, our DTI study provides evidence that adults with PKU show alterations in WM microstructure, and that these alterations are particularly prominent in posterior regions of the brain. Our findings further suggest an association between worse cognitive performance and DTI metrics, whereas WM lesion load was unrelated to cognition in adults with PKU. Hence, DTI adds value to the understanding of the interplay between cognition and WM microstructure in adults with PKU.

## Supplementary Material

fcad155_Supplementary_Data

## Data Availability

Upon reasonable request and with the consent of the study team, the study data are available from the corresponding author.

## Abbreviations

AD =: axial diffusivity
ANTs =: Advanced Normalization Tools
CI =: confidence interval
D-KEFS =: Delis–Kaplan Executive Function System
DTI =: diffusion tensor imaging
EPI =: echo planar imaging
FA =: fractional anisotropy
FDR =: false discovery rate
hCs =: homocarnosine
IDC =: index of dietary control
IQR =: interquartile range
max =: maximum value
MD =: mean diffusivity
min =: minimum value
MPRAGE =: Magnetization Prepared-RApid Gradient Echo
NAA =: *n*-acetylaspartate
*P* =: *P*-value
Phe =: phenylalanine
PICO =: Phenylalanine and its Impact on COgnition study
PKU =: phenylketonuria
RD =: radial diffusivity
ROI =: region of interest
*r*_rb_ =: rank-biserial correlation coefficient
*r*_s_ =: Spearman’s rank correlation coefficient
TA =: acquisition time
TAP =: Test of Attentional Performance
TBSS =: tract-based spatial statistics
TE =: echo time
TI =: inversion time
TR =: repetition time
Trp =: tryptophan
Tyr =: tyrosine
U =: Mann–Whitney U statistic
VAPOR =: variable power and optimized relaxation delays
WAIS-IV =: Wechsler Adult Intelligence Scale Fourth Edition
WM =: white matter
