# Supplementary material for: Compromised white matter is related to lower cognitive performance in adults with phenylketonuria
Source: Brain Commun. 2023 May 15;5(3):fcad155. doi: 10.1093/braincomms/fcad155 (PMC10231812; doi:10.1093/braincomms/fcad155)
Supplement: fcad155_Supplementary_Data [file fcad155_supplementary_data.pdf]

Supplement

Supplementary Table 1. Demographics of patients and controls.

		**Patients (n=30)**	**Controls (n=54)**	U/χ^2	p	r_{rb} / φ
Age	median (IQR)	35.5 years (12.3)	29.3 years (9.4)	943.0	0.216	0.16
	range	19-48 years	18-53 years			
Sex	female	13	26	0.0	0.845	0.30
	male	17	27			
Education	median (IQR)	6.0 (2.8)	5.0 (3.8)	4.6	0.707	0.23
IQ	median (IQR)	97.0 (16.0)	109.0 (20)	498	0.004	-0.39

All values were gathered at the time of the MRI. Abbreviations: IQR= interquartile range; U=Mann-Whitney U-statistic; χ^2=Chi-squared statistic; effect sizes are reported as rank-biserial correlations (r_{rb}) for Mann-Whitney U-tests and as phi (φ) for chi-squared tests; education was categorized into 1=Secondary Education, 2=Apprenticeship, 3=Vocational Education, 4=High School, 5=College of Higher Education, 6=Bachelor or equivalent, 7=Master or equivalent, 8=Doctorate.

Supplementary Table 2. Clusters of significant group difference in FA, MD, and AD between patients with PKU and healthy controls

DTI metric	Anatomical location	Center of gravity (mm)			Cluster size	Max T	$P_{FWE\text{-}corr}$
		x	*y*	*z*			
FA	Posterior corona radiata R[1] & middle longitudinal fasciculus R[2]	30	-39.7	19.2	637	4.71	<0.001
	Posterior thalamic radiation L[1] & middle longitudinal fasciculus L[2]	-32.5	-45.2	15.1	502	4.75	<0.001
	Body CC[1]	13.6	-22.7	28.6	490	5.06	<0.001
	SLF II L[2]	-32.2	-39.1	33.5	381	4.68	<0.001
	Forceps major[2]	-28.7	-71.4	9.37	252	3.74	<0.001
	Body CC[1]	-13.5	-19.5	30.8	181	4.69	<0.001
	Uncinate fasciculus L[2]	-36.1	-4.84	-19.9	151	4.13	<0.001
	Anterior corona radiata R[1] & anterior thalamic radiation R[2]	23.3	33	12.1	142	4.37	<0.001
	Genu CC[1] & forceps minor[2]	7.26	28.3	-0.02	135	4.3	<0.001
	Genu CC[1] & forceps minor[2]	9.21	24.8	15.1	119	3.8	<0.001
	Superior thalamic radiation L[2]	-14.1	2.36	54.7	116	4.6	<0.001
	Anterior corona radiata L[1]	-17.8	21.8	29.1	109	3.51	<0.001
	Anterior corona radiata R[1] & forceps minor[2]	19.9	37.5	12.5	105	3.51	<0.001
	Optic radiation L[2]	-21.6	-84	3.64	100	4.27	<0.001
	Middle longitudinal fasciculus L[2]	-26	-69.5	22	99	4.82	<0.001
	Frontal aslant tract L[2]	-18.3	17.3	36.1	98	3.51	<0.001
	SLF II R[2]	29.9	-34.8	38.5	92	3.44	<0.001
	Near SLF I R[2]	18	-57.6	36.2	90	3.75	<0.001
	Anterior corona radiata R[1] & Inferior fronto-occipital fasciculus R[2]	25.9	20.5	5.58	86	3.62	<0.001
	Anterior corona radiata L[1] & forceps minor[2]	-21.2	32.7	12.5	85	3.86	<0.001
	SLF R[1] & arcuate fasciculus R[2]	36.6	-39.3	27.6	83	4.24	<0.001
	Superior corona radiata L[1] & frontal aslant tract L[2]	-23.6	10.7	32.8	79	3.54	<0.001
	Inferior longitudinal fasciculus L[2]	-45.2	-17	-19.2	78	3.75	<0.001
	Superior corona radiata L[1] & frontal aslant tract L[2]	-25.1	8.35	19.5	71	3.96	<0.001
	Superior thalamic radiation R[2]	25.3	-6.03	18.5	64	3.57	<0.001
	Sagittal stratum[1] & inferior longitudinal fasciculus L[2]	-40.8	-31.5	-13.5	55	3.79	<0.001
	Posterior thalamic radiation R[1] & inferior fronto-occipital fasciculus R[2]	38.9	-41.9	-2.19	51	3.43	0.002
MD	Body CC[1]	0.17	-29	20.3	23741	13.2	<0.001
	Anterior corona radiata R[1] & anterior thalamic radiation R[2]	24.8	33.5	6.74	555	9.31	<0.001
	Near forceps major[2]	22.6	-46.8	3.76	156	5.63	<0.001
	SLF R[1] & frontal aslant tract R[2]	38.1	6.03	22.8	122	5.01	<0.001
	Middle longitudinal fasciculus L[2]	-31.3	-50.5	25.4	78	11.5	<0.001
	Splenium CC[1] & forceps major[2]	-16.1	-42.5	8.45	60	4.99	0.001
	Fornix L[2]	-38.2	-7.45	-22.3	58	3.57	0.002
AD	Body CC[1]	-1.29	-29.9	19.3	20801	14.2	<0.001
	SLF R[1] & arcuate fasciculus R[2]	37.3	-42.3	22.6	1175	6.75	<0.001
	Anterior corona radiata R[1] & anterior thalamic radiation R[2]	24.2	33.2	8.68	390	8.94	<0.001
	Uncinate fasciculus L[2]	-35.6	-5.97	-18.1	290	5.77	<0.001
	Anterior corona radiata L[1] & anterior thalamic radiation L[2]	-25.7	23.1	16.6	254	6.01	<0.001
	Superior corona radiata R[1] & superior thalamic radiation R[2]	23.8	-9.29	35.5	207	7.23	<0.001
	Frontal aslant tract R[2]	26	24	26.1	177	5.16	<0.001
	SLF I L[2]	-14.1	3.21	54.6	148	4.39	<0.001
	Near inferior fronto-occipital fasciculus R[2]	37.4	-5.55	-21.4	134	5.68	<0.001
	Anterior thalamic radiation L[2]	-19.5	47.7	7.28	85	3.7	<0.001
	Middle longitudinal fasciculus L[2]	-31.3	-50.5	25.2	78	12.3	<0.001
	Posterior limb of the internal capsule R[1] & superior thalamic radiation R[2]	25.4	-6.23	18.5	76	4.7	<0.001
	Forceps major[2]	-23.1	-47.6	2.75	70	6.0	<0.001
	SLF R[1] & SLF III R[2]	35	8.73	23.2	61	3.65	0.001
	Splenium CC[1] & forceps major[2]	-15.4	-41.5	8.25	60	5.53	0.001
	Anterior thalamic radiation L[2]	-26.5	38.2	1.32	60	4.81	0.001
	Anterior commissure[2]	29.6	-5.82	-13.3	53	4.15	0.005

[1]JHU labels, [2]XTRACT

Abbreviations: AD, axial diffusivity; CC, corpus callosum; FA, fractional anisotropy; FWE, family-wise error correction; L, left; MD, mean diffusivity; *P*, *P*-value; SLF, superior longitudinal fasciculus; R, right.

Supplementary Table 3. Cognitive performance in patients and controls

			Patients (n=30) Median (IQR)	Controls (n=54) Median (IQR)	*U*	*P*	r_{rb}	[95% CI] for r_{rb}
**Processing speed**	Processing speed	Scale scores[1]	10.0 (3.0)	12.0 (3.0)	1155	0.001*	0.43	[0.19, 0.61]
		Residuals[1]	-5.2 (9.7)	0.4 (9.5)	1140	0.002*	0.41	[0.17, 0.60]
**Executive functions**	Working memory	Raw scores (accuracy in %)[1]	94.5 (7.0)	98.0 (3.0)	478	0.002*	0.41	[0.17, 0.60]
		Residuals[1]	-2.4 (7.0)	0.8 (3.2)	490	0.003*	0.40	[0.16, 0.59]
	Inhibition	Scale scores (time)[1]	10.0 (2.5)	11.0 (3.8)	654	0.143	0.19	[-0.06, 0.43]
		Residuals[2]	0.1 (13.7)	-0.7 (11.5)	931	0.261	-0.15	[-0.39, 0.11]
	Cognitive flexibility	Scale scores (time)[1]	10.0 (2.0)	12.0 (3.0)	457	0.001*	0.44	[0.21, 0.62]
		Residuals[2]	6.4 (10.2)	-2.30 (11.8)	1175	0.001*	-0.45	[-0.63, -0.22]
**Attention**	Alertness	T-scores (median reaction time)[1]	45.5 (9.8)	47.0 (12.0)	672	0.199	0.17	[-0.09, 0.41]
		Residuals[2]	3.2 (37.4)	-0.8 (32.7)	899	0.409	-0.11	[-0.35, 0.15]
	Divided attention	T-scores (total omissions)[1]	48.0 (13.0)	53.0 (13.0)	698	0.418	0.11	[-0.15, 0.35]
		Residuals[2]	-0.05 (1.9)	-0.6 (2.1)	978	0.063	-0.25	[-0.47, 0.01]
	Sustained attention	T-scores (sd in reaction time)[1]	46.0 (9.0)	52.0 (9.0)	453	0.002*	0.41	[0.17, 0.60]
		Residuals[2]	28.5 (48.8)	-9.4 (50.4)	1100	0.003*	-0.40	[-0.60, -0.17]

Abbreviations: IQR=interquartile range; *U*=Mann-Whitney *U*-statistic; *P*=*P*-value; effect sizes are reported as rank-biserial correlations (r_{rb}) for Mann-Whitney *U*-tests; CI=confidence interval; sd=standard deviation; residuals are the error terms after regressing out the confounding variable age; [1]the higher/the more positive the score, the better performance, [2] the more negative the score, the better performance, * survives FDR-correction.

Supplementary Table 4. Significant correlations between DTI metrics and metabolic parameters

Metabolic parameter	DTI metric and ROI	r_s	*P*	95% CI
Phe (blood)	FA anterior limb of the internal capsule	0.37	0.044	0.01, 0.67
	FA external capsule	0.38	0.040	-0.01, 0.69
	MD anterior limb of the internal capsule	−0.40	0.028	−0.69, −0.08
	MD external capsule	−0.37	0.042	−0.65, −0.00
Phe (brain)	MD external capsule	−0.37	0.047	−0.65, 0.02
	MD optic radiation	−0.39	0.033	−0.68, 0.02
Tyr	FA optic radiation	0.42	0.023	0.09, 0.66
	FA superior longitudinal fasciculus	0.37	0.045	0.03, 0.66
	FA splenium of the corpus callosum	0.40	0.027	0.04, 0.71
IDC 0–5	FA anterior limb of the internal capsule	0.50	0.040	0.01, 0.80
IDC 6–12	FA external capsule	0.61	0.007	0.13, 0.92
	FA posterior limb of the internal capsule	0.52	0.024	0.05, 0.82
	FA superior corona radiata	0.55	0.016	0.09, 0.85
IDC 18+	FA anterior limb of the internal capsule	0.54	0.023	0.08, 0.87
	FA anterior corona radiata	0.57	0.015	0.05, 0.89
	FA external capsule	0.56	0.017	0.04, 0.88
	FA posterior corona radiata	0.53	0.025	−0.01, 0.85
	MD external capsule	−0.53	0.026	−0.75, −0.16
IDC lifetime	FA anterior limb of the internal capsule	0.58	0.032	0.02, 0.89
	FA anterior corona radiata	0.65	0.015	0.05, 0.96
	FA external capsule	0.75	0.003	0.30, 0.96
	FA genu of the corpus callosum	0.55	0.044	−0.05, 0.95
	FA posterior limb of the internal capsule	0.61	0.023	0.17, 0.85
	FA posterior corona radiata	0.63	0.019	0.06, 0.93
	MD external capsule	−0.69	0.008	−0.92, −0.26
	MD inferior longitudinal fasciculus	−0.61	0.023	−0.84, −0.14

Abbreviations: CI, confidence interval; DTI, diffusion tensor imaging; FA, fractional anisotropy; IDC, index of dietary control; MD, mean diffusivity; *P*, *P*-value; Phe, phenylalanine; r_s, Spearman's rank correlation coefficient; Tyr, tyrosine.

Note: No correlation survived FDR correction.

Supplementary Table 5. Significant correlations between DTI metrics and cognitive performance (PATIENTS)

Cognitive task	DTI metric and ROI	r_s	*P*	95% CI
Processing speed	FA body of the corpus callosum	0.46	0.012	0.13, 0.71
	FA external capsule	0.45	0.013	0.08, 0.74
	FA inferior longitudinal fasciculus	0.41	0.027	0.07, 0.67
	FA posterior corona radiata	0.39	0.035	0.05, 0.65
Inhibition	FA posterior corona radiata	−0.42	0.021	−0.67, −0.08
	MD anterior corona radiata	−0.48	0.008	−0.76, −0.12
	MD genu of the corpus callosum	−0.43	0.018	−0.69, −0.07
	MD external capsule	−0.41	0.027	−0.69. −0.01
	MD inferior longitudinal fasciculus	−0.52	0.004	−0.76, −0.20
	MD optic radiation	−0.43	0.017	−0.71, −0.08
	MD posterior corona radiata	−0.38	0.038	−0.67, −0.01
	MD splenium of the corpus callosum	−0.55	0.002	−0.71, −0.29
	MD superior corona radiata	−0.53	0.003	−0.71, −0.27
	MD superior longitudinal fasciculus	−0.55	0.002	−0.77, −0.22
	AD anterior corona radiata	−0.54	0.002	−0.79, −0.17
	AD genu of the corpus callosum	−0.38	0.037	−0.69, −0.04
	AD posterior corona radiata	−0.52	0.004	−0.73, −0.22
	AD splenium of the corpus callosum	−0.44	0.016	−0.65, −0.12
	AD superior corona radiata	−0.56	0.002	−0.75, −0.25
Alertness	AD external capsule	−0.38	0.037	−0.66, −0.00
Divided attention	FA body of the corpus callosum	−0.47	0.010	−0.73, −0.15
	FA inferior longitudinal fasciculus	−0.50	0.007	−0.74, −0.12
	MD external capsule	0.44	0.019	0.08, 0.71
	MD inferior longitudinal fasciculus	0.38	0.044	−0.02, 0.73

Abbreviations: AD, axial diffusivity; CI, confidence interval; DTI, diffusion tensor imaging; FA, fractional anisotropy; MD, mean diffusivity; *P*, *P*-value; r_s, Spearman's rank correlation coefficient.
Note: No correlation survived FDR correction. The higher the scores for processing speed, the better the performance. The lower the scores for inhibition, alertness, and divided attention, the better the performance.

Supplementary Table 6. Significant correlations between DTI metrics and cognitive performance (CONTROLS)

**Cognitive task**	**DTI metric and ROI**	r_s	***P***	**95% CI**
Processing speed	MD body of the corpus callosum	0.27	0.045	0.04, 0.50
Working memory	FA external capsule	0.33	0.016	0.07, 0.55
	AD optic radiation	0.34	0.011	0.08, 0.57
	AD external capsule	0.34	0.014	0.07, 0.56
Inhibition	FA external capsule	-0.43	0.001	-0.64, -0.19
	AD body of the corpus callosum	-0.30	0.029	-0.53, -0.04
	AD splenium of the corpus callosum	-0.29	0.034	-0.53, -0.03
Cognitive flexibility	FA external capsule	-0.28	0.040	-0.52, -0.02
Alertness	MD posterior limb of the internal capsule	-0.30	0.029	-0.55, -0.03
	AD anterior corona radiata	-0.30	0.029	-0.53, -0.03
Divided attention	FA posterior limb of the internal capsule	-0.33	0.014	-0.54, -0.90
	MD body of the corpus callosum	-0.34	0.011	-0.60, -0.05
	MD genu of the corpus callosum	-0.33	0.017	-0.58, -0.02
	AD genu of the corpus callosum	-0.36	0.007	-0.60, -0.11
	AD posterior corona radiata	-0.29	0.033	-0.54, -0.03
	AD posterior limb of the internal capsule	-0.29	0.032	-0.53, -0.02

Abbreviations: AD, axial diffusivity; CI, confidence interval; DTI, diffusion tensor imaging; FA, fractional anisotropy; MD, mean diffusivity; *P*, *P*-value; r_s, Spearman's rank correlation coefficient.

Note: No correlation survived FDR correction. The higher the scores for processing speed and working memory, the better the performance. The lower the scores for inhibition, cognitive flexibility, alertness, and divided attention, the better the performance.
